# Attenuation of Oxidative Stress and Regulation of AKT Signaling by Vanillic Acid during Bovine Pre-Implantation Embryo Development

**DOI:** 10.3390/nu15102257

**Published:** 2023-05-10

**Authors:** Marwa El-Sheikh, Ayman Mesalam, Myeong-Don Joo, Tabinda Sidrat, Ahmed Atef Mesalam, Il-Keun Kong

**Affiliations:** 1 Department of Microbial Biotechnology, Biotechnology Research Institute, National Research Centre (NRC), Dokki, Cairo 12622, Egypt; marwa.el-sheikh@hotmail.com; 2 Department of Theriogenology, Faculty of Veterinary Medicine, Zagazig University, Zagazig 44519, Egypt; aymanmesalam@gmail.com; 3 Division of Applied Life Science (BK21 Four), Gyeongsang National University, Jinju 52828, Republic of Korea; jmd1441@gmail.com (M.-D.J.); tabindasidrat06@gmail.com (T.S.); 4 Department of Therapeutic Chemistry, Pharmaceutical and Drug Industries Research Institute, National Research Centre (NRC), Dokki, Cairo 12622, Egypt; 5 Institute of Agriculture and Life Science, Gyeongsang National University, Jinju 52828, Republic of Korea; 6 The King Kong Corp. Ltd., Gyeongsang National University, Jinju 52828, Republic of Korea

**Keywords:** vanillic acid, oxidative stress, AKT signaling, oocyte, embryo

## Abstract

Vanillic acid (VA) has shown antioxidant and anti-inflammatory activities in different cell types, but its biological effects in the context of early embryo development have not yet been clarified. In the current study, the impact of VA supplementation during in vitro maturation (IVM) and/or post-fertilization (in vitro culture; IVC) on redox homeostasis, mitochondrial function, AKT signaling, developmental competence, and the quality of bovine pre-implantation embryos was investigated. The results showed that dual exposure to VA during IVM and late embryo culture (IVC3) significantly improved the blastocyst development rate, reduced oxidative stress, and promoted fatty acid oxidation as well as mitochondrial activity. Additionally, the total numbers of cells and trophectoderm cells per blastocyst were higher in the VA-treated group compared to control (*p* < 0.05). The RT-qPCR results showed down-regulation of the mRNA of the apoptosis-specific markers and up-regulation of *AKT*2 and the redox homeostasis-related gene *TXN* in the treated group. Additionally, the immunofluorescence analysis showed high levels of pAKT-Ser473 and the fatty acid metabolism marker CPT1A in embryos developed following VA treatment. In conclusion, the study reports, for the first time, the embryotrophic effects of VA, and the potential linkage to AKT signaling pathway that could be used as an efficacious protocol in assisted reproductive technologies (ART) to improve human fertility.

## 1. Introduction

Tight regulation of various molecular and physiological processes is crucial for the optimal in vitro and in vivo development of oocytes and pre-implantation embryos [1]. For instance, the redox state represents a critical mechanism for maintenance of normal cell function ensuring the normal oocyte maturation, blastocyst development, hatching, and implantation [2,3]. However, redox imbalance can impair embryo development, causing transgenerational effects on metabolism, genome function, and postnatal progeny health [1,2].

Reactive oxygen species (ROS) were first described as byproducts of ATP generation mediated by mitochondrial respiration. The respiratory chain process is a major source of ROS, whereas mitochondrial dysfunction is mainly associated with high ROS levels that cause damage to mitochondrial DNA, proteins, and lipids, and the high levels contribute to wide ranges of irreversible oxidative injury, thereby disrupting the overall mitochondrial function [4,5]. Under normal conditions, the mitochondrial antioxidant system can maintain redox homeostasis by controlling the cellular ROS at certain levels for cell survival, activating key signals, proliferation, and differentiation [1,4,6]. In assisted reproductive technologies (ART), ROS accumulation has been recognized as one of the main barriers for well-developed embryos [2]. Thus, understanding the molecular mechanisms regulating the process of oxidative stress has encouraged the potential application of several bioactive compounds for diminishing the pressure of ROS.

During the process of oocyte maturation, a wide range of enzymes have been reported to play essential roles in various cellular events [2,7]. The available literature suggests that oocytes’ metabolic activity may be a key target mediating several reproductive defects [8]. AKT, a serine/threonine specific protein kinase, plays a key role in cell proliferation [9]. The phosphatidylinositol-3-kinase (PI3K)/AKT pathway is also associated with ovarian function, including oocyte maturation, developmental competence of embryos, and granulosa cell proliferation [10,11,12]. On the other hand, vanillic acid (VA) is a flavoring agent with antioxidant and anti-inflammatory properties. Additionally, it exhibits antimicrobial, anti-obese, anti-diabetic, antitumor, and cytoprotective activities [13,14,15,16,17,18]. A growing body of literature suggests that VA can scavenge and remove ROS radicals. The protective effect of VA against ROS in drosophila line 2 (d.mel-2) cells was previously reported [18]. Additionally, it has been stated that VA increases the levels of phosphorylated AKT (pAKT) in dermal papilla cells (DPCs) in a time-dependent manner [19]. In the same study, the inhibition of the PI3K/AKT pathway, using wortmannin, attenuated VA-induced proliferation [19]. In another study, VA administration abrogated the neurotoxicity of amyloid beta, the main contributor to Alzheimer’s disease, in both neuronal HT22 cells and mice through up-regulating pAKT and attenuating the oxidative stress [17]. In addition, injection of mice with VA displayed an antidepressant effect in an AKT-dependent manner; a process that was neutralized by the AKT blocker MK2206 [20].

Although the protective effect of VA against ROS, as well as the interplay between VA and AKT signaling, have been reported in several cell types [17,18,19,20], the potential effect and interaction in oocytes and embryos are still unknown. Hereby, in the current study, we used VA to first check its effect on the developmental competence of bovine oocytes and subsequent embryo development. Moreover, we clarified its impact when supplemented at IVM and/or late embryo culture (IVC3) on the developmental competence of oocytes and embryos. The potential interplay between VA and AKT signaling was also investigated.

## 2. Materials and Methods

### 2.1. Experimental Design

Four experimental set-ups were executed in the study: Experiment 1 was performed to study the effect of VA supplementation, to in vitro maturation (IVM) medium, on the developmental competence of embryos. In this experiment, four different concentrations (2, 20, 200, and 2000 µM) of VA (Sigma-Aldrich, St. Louis, MO, USA) were used, while the total cleavage, 8–16 cell-stage embryos, and day-8 blastocyst development rates were recorded. Based on the results of the first set-up, experiments 2 and 3 were performed using 2 µM of VA either as a single shot at different stages of in vitro culturing (IVC1, 2 or 3), or a dual treatment during IVM and IVCs, whereas the day-8 blastocyst development rate was investigated. Based on these experiments, experiment 4 was carried out where two groups corresponding to an untreated control and the dual treatment group (2 µM of VA during IVM and late in vitro culturing (IVC3)) were included. In this experiment, day-8 blastocysts were collected and used for RT-qPCR, immunofluorescence, and estimation of intracellular ROS levels and mitochondrial activity. Experiments were conducted according to the regulations of the Institutional Animal Care and Use Committee (IACUC) of Gyeongsang National University (Approval ID: GAR-110502-X0017).

### 2.2. In Vitro Maturation (IVM) of Oocytes

Cumulus–oocyte complexes (COCs) were aspirated from antral follicles (2–8-mm-diameter) of slaughtered Hanwoo cows’ ovaries, using 18-gauge needles and washed 3 times in TL-HEPES (10 mM HEPES, 114 mM sodium chloride, 3.2 mM potassium chloride, 0.5 mM magnesium chloride, 2 mM calcium chloride, 0.34 mM sodium biphosphate, 2 mM sodium bicarbonate, 10 mM sodium lactate, 1 μL/mL phenol red, 0.1 mg/mL streptomycin, and 100 IU/mL penicillin). Groups of 50 COCs (with at least 3 layers of cumulus cells) were then cultured in 4-well dishes containing 500 μL of IVM medium (TCM-199 supplemented with 10 μg/mL FSH, 1 μg/mL estradiol-17β, 10 ng/mL EGF, 0.2 mM sodium pyruvate, 0.6 mM cysteine, 0.1 mg/mL streptomycin, and 100 IU/mL penicillin), in the presence or absence of different VA concentrations (dissolved in ethanol and further diluted in IVM medium), and incubated for 22–24 h at 38.5 °C under 5% CO_2_.

### 2.3. In Vitro Fertilization (IVF)

Frozen Hanwoo semen was thawed at 37 °C, washed in pre-warmed Dulbecco’s phosphate-buffered saline (DPBS), and centrifuged at 1800 rpm for 5 min. Sperm pellet was then diluted in IVF medium (Tyrode’s lactate solution supplemented with 20 μg/mL heparin, 6 mg/mL BSA, 22 µg/mL sodium pyruvate, 0.1 mg/mL streptomycin, and 100 IU/mL penicillin) to a final concentration of 1 × 10^6^ spermatozoa/mL. Each group of in vitro maturated oocytes was then loaded with 500 μL of spermatozoa followed by incubation at 38.5 °C and 5% CO_2_ for 18–20 h.

### 2.4. In Vitro Culture (IVC)

Following co-incubation with spermatozoa, COCs were repetitively pipetted to remove the cumulus cells, while groups of 50 presumed zygotes were maintained in 500 μL of IVC medium, in the presence or absence of VA, and incubated at 38.5 °C (IVC1). The medium was replenished, and VA was added at day 4 or day 6 post-fertilization (corresponding to IVC2 and IVC3, respectively; the day of fertilization was considered as day 0). The total cleavage and the number of 8–16 cell-stage embryos were recorded at day 4 post-fertilization while blastocyst development was calculated at day 8 post-fertilization. Blastocysts were either fixed in 4% paraformaldehyde for use in staining experiments or kept at −80 °C for use in RNA extraction.

### 2.5. Trophectoderm (TE) and Inner Cell Mass (ICM) Counting

Differential staining of TE and ICM cells was performed as previously stated [21]. In brief, fixed blastocysts (20 per group) were washed in DPBS and permeabilized with 0.25% Triton X-100 for 20 min at room temperature. Embryos were blocked for 1 h at room temperature before being incubated overnight at 4 °C with an anti-caudal-related homeobox 2 (CDX2) antibody (BioGenex, Hague, Netherlands). After washing in DPBS, embryos were incubated with Alexa Fluor-568 donkey anti-mouse IgG (1:500; Invitrogen/Molecular Probes, Eugene, OR, USA, A10037) for 60 min at room temperature; then, the nuclei were stained with 4’,6-diamidino-2-phenylindole (DAPI, 1 µg/mL) for 15 min. Embryos were washed and mounted on glass slides and examined under a confocal laser-scanning microscope (Olympus, Tokyo, Japan) at 405 nm excitation and 461 nm emission for DAPI and 559 nm excitation and 603 nm emission for Alexa Fluor-568. The total number of cells were enumerated by counting the DAPI positive cells, while TE and ICM cells were examined through recording the CDX2 positive and negative cells, respectively. Additionally, the ICM:TE ratio was calculated by dividing the ICM/TE cells.

### 2.6. Quantification of Intracellular ROS Levels and Cytoplasmic Lipid Content

Day-8 blastocysts (20 per group) were incubated with 5 μM of the ROS indicator 2,7-dichlorodihydrofluorescein diacetate (H_2_DCFDA, D6883) for 20 min at 38.5 °C and then directly imaged under an epifluorescence microscope (Olympus IX71, Tokyo, Japan) at 488 nm excitation and 519 nm emission. Quantification of fluorescence intensities was performed using ImageJ (National Institutes of Health, Bethesda, USA; https://imagej.nih.gov/ij, accessed on 9 March 2023). For cytoplasmic lipid content, fixed blastocysts were stained with Nile red fluorescent probe (10 µg/mL) for 3 h at room temperature while the nuclei were stained with DAPI for 15 min, followed by washing with DPBS and mounting onto glass slides. A confocal laser-scanning microscope was used to excite the lipophilic fluorescent dye at 559 nm excitation and 581 nm emission whereas the fluorescence intensities were estimated in relation to the total number of nuclei per blastocyst.

### 2.7. Estimation of Mitochondrial Membrane Potential (ΔΨ_m_)

For mitochondrial membrane potential, blastocysts (20 per group) were stained with JC-1 fluorescent probe (2 μg/mL; Invitrogen/Molecular Probes, T3168) for 30 min and spotted on glass slides after being washed with DPBS. Images were captured with a confocal laser scanning microscope at 488 nm excitation and 520 nm emission, whereas the fluorescence intensities of JC-Monomer (green, low membrane potential) and JC-aggregate (red, high membrane potential) were estimated using ImageJ. The ΔΨ_m_ represents the ratio of JC-aggregate versus JC-monomers.

### 2.8. RT-qPCR

Total RNA was extracted from oocytes and blastocysts (50 and 5, respectively; 4 biological replicates) using an Arcturus PicoPure RNA isolation kit according to the manufacturer’s guidelines (Arcturus, Foster, CA, USA) and reverse-transcribed using the iScript cDNA synthesis kit (Bio-Rad Laboratories, Hercules, CA, USA). The qPCR was performed using a CFX96 instrument (Bio-Rad Laboratories, Hercules, CA, USA) using iQ-SYBR Green Supermix kit (Bio-Rad Laboratories) where the following conditions were applied: initial denaturation at 95 °C for 3 min followed by 44 cycles of 95 °C for 15 s, 58 °C for 20 s, and 72 °C for 30 s. The sequences of the used primers are listed in Table 1. The expressions of different genes were relatively quantified via the 2^−ΔΔCt^ method where *GAPDH* was used as a reference gene.

### 2.9. Immunofluorescence

Fixed blastocysts (20 per group) were washed in DPBS and permeabilized with 0.5% Triton X-100 for 20 min at room temperature. Embryos were blocked (10% FBS and 3% BSA) for 2 h at room temperature followed by overnight incubation at 4 °C with antibodies raised against CPT1A (1:200; Santa Cruz Biotechnology, Santa Cruz, CA, USA) and pAKT-Ser473 (1:200; Cell Signaling, Danvers, MA, USA). Samples were washed 3 times in DPBS and incubated at room temperature for 90 min with the Alexa Fluor-568 donkey anti-mouse IgG (1:500; Thermo Fisher Scientific, Waltham, MA, USA, A10037), and Alexa Fluor-488 donkey anti-rabbit IgG antibodies (1:500; Thermo Fisher Scientific, A-11055). After staining the nuclei with DAPI, embryos were mounted on glass slides and investigated under a confocal laser scanning microscope at 559 nm excitation and 603 nm emission for Alexa Fluor-568, and 488 nm excitation and 520 nm emission for Alexa Fluor-488, while quantification of fluorescence intensities was performed using ImageJ.

### 2.10. Statistical Analysis

The statistical analysis was performed using GraphPad Prism version 6 (GraphPad Software, San Diego, CA, USA). The differences in embryonic development were analyzed via one-way ANOVA, whereas blastocyst quality and gene expression were analyzed using Student’s *t*-test. The data in the current study conformed to normal distribution, and are presented as the mean values ± standard error of the mean (SEM). *p* values below 0.05 were considered statistically significant.

## 3. Results

### 3.1. Effect of VA Administration on Oocyte and Pre-Implantation Embryo Development

**Figure 1:**
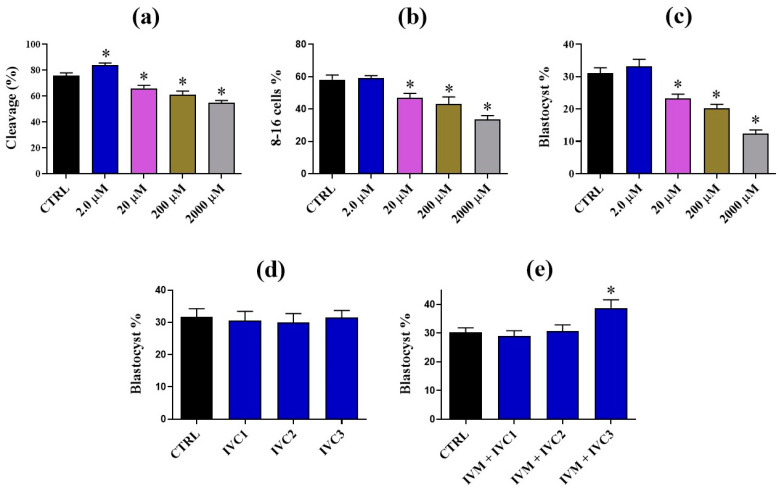
Effect of vanillic acid (VA) on the developmental competence of bovine oocytes and embryos. (**a**) Cleavage rates post VA supplementation during IVM. (**b**) The 8–16 cell stage embryos after IVM in presence and absence of VA. (**c**) The percentages of the developed embryos after VA administration during IVM. (**d**) Effect of VA (2 µM) added post fertilization (IVC1, 2, or 3) on the blastocyst development. (**e**) Blastocyst development rates after dual treatment of VA (IVM + IVC1, 2 or 3). *p* value < 0.05 was reported a statistically significance and presented as asterisk (*).

To evaluate the regulatory role of VA, supplemented during the process of in vitro maturation (IVM), on oocytes, we first sought to check the competency of embryo development, a process that can reflect the quality of oocyte. The results demonstrated that low VA concentration (2 µM) during IVM significantly enhanced the total number of cleaved embryos (84 ± 1.58% vs. 75.8 ± 2.20% for control; *p* < 0.05) without affecting the rates of 8–16 cell-stage embryos and day-8 blastocyst development (Figure 1a–c). Contrarily, a significant reduction was detected in all developmental parameters when VA was supplemented at high concentrations (above 2 µM; Figure 1a–c).

In a similar experimental setting, the impact of VA treated during IVM and/or different stages of in vitro culture (IVC1, 2, or 3) was estimated. Although the sole treatment of VA during IVC did not show any effect on blastocysts (*p* > 0.05; Figure 1d), the dual treatment during IVM and the late stage of culturing (IVC3) significantly improved the development rate (38.75 ± 2.87% vs. 30.25 ± 1.60% for control; *p* < 0.05; Figure 1e).

**Figure 2:**
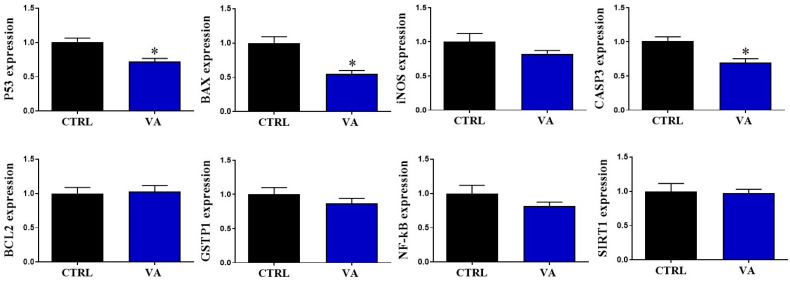
Relative expression of the mRNA of genes involved in oxidative stress and redox homeostasis in oocytes post VA supplementation. *p* value < 0.05 was reported a statistically significance and presented as asterisk (*).

Due to the cytoprotective effect of VA, previously reported in mammalian cells [14,16,17], we investigated the effect of VA on the quality of bovine oocytes at the molecular level. Specific markers regulating redox homeostasis and oxidative stress, such as *P53*, *BAX*, *CASP3*, *iNOS*, *BCL2*, *NF-kB*, *SIRT1*, and *GSTP1*, were tested in oocytes using RT-qPCR. Among the tested genes, a significant down-regulation was observed in the apoptosis-related genes *P53*, *BAX*, and *CASP3* upon VA treatment compared to the untreated oocytes (Figure 2).

### 3.2. Beneficial Effects of VA on the Quality of Blastocysts

**Figure 3:**
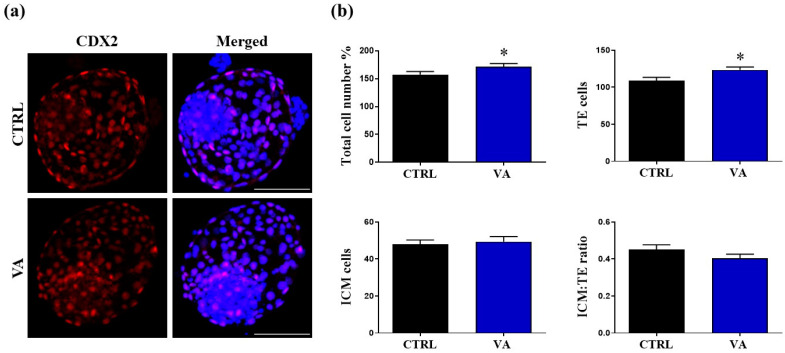
Differential staining of blastocysts following VA treatment. (**a**) Representative images of day-8 blastocysts stained with DAPI (blue) and CDX2 (red). Scale bar, 100 µm. (**b**) Total number of cells, trophectoderm, ICM and the ICM:TE ratio per blastocyst. *p* value < 0.05 was reported a statistically significance and presented as asterisk (*).

Counting the blastocyst inner cell mass (ICM) and trophectoderm (TE) cells have been used as indicators of cultured blastocyst quality, known as the CDX2-based differential staining assessment [21,22]. As shown in Figure 3, the numbers of the total cells and TE cells per blastocyst were significantly higher in the VA-supplemented group (171.32 ± 5.97 and 123.42 ± 3.93, respectively) than the untreated control (157.47 ± 5.38 and 108.68 ± 4.59, respectively). Although the ICM:TE ratio was slightly lower in the VA-treated group than the control, this difference did not reach statistical significance (*p* > 0.05; Figure 3).

**Figure 4:**
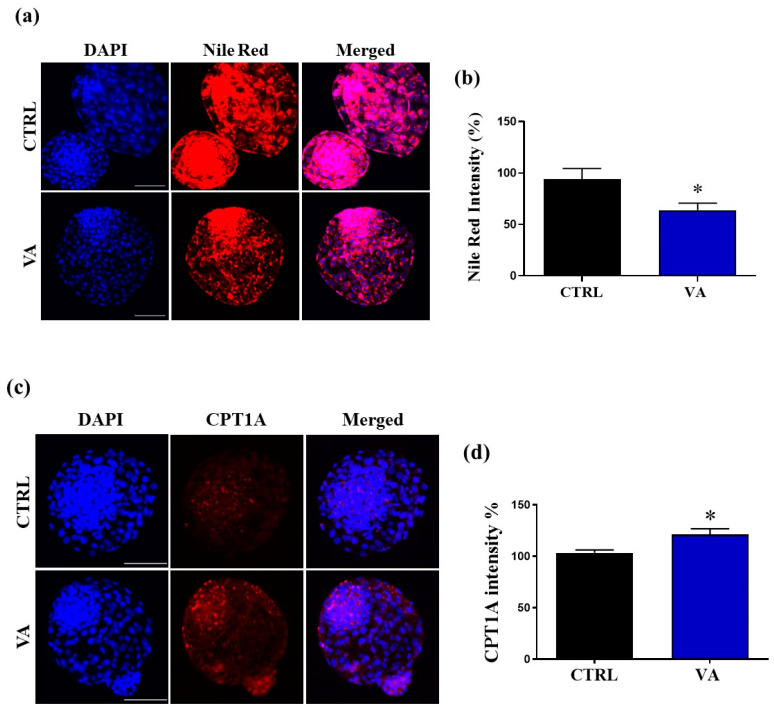
Effect of VA on lipid metabolism. (**a**) Representative images of lipid droplets accumulated in day-8 blastocyst using Nile red staining. (**b**) Integrated optical intensity of Nile red staining in relation to DAPI nuclei number in VA-treated and control groups. (**c**) Representative images of embryos stained with CPT1A, the fatty acid metabolism marker. Scale bar, 100 µm. (**d**) Fluorescence intensity of CPT1A in blastocysts of control and VA-treated groups. *p* value < 0.05 was reported a statistically significance and presented as asterisk (*).

To test the beneficial effects of VA on lipid metabolism, we evaluated lipid droplet accumulation using Nile red staining during blastocyst development. The integrated optical intensity measured by ImageJ revealed that the accumulated lipid content was significantly (*p* < 0.05) lower in the VA-treated group as compared to control (Figure 4a,b). We further measured the expression of carnitine palmitoyltransferase 1A (CPT1A), the lipid metabolism-related marker [23], using immunofluorescence. Cpt1A is a critical factor for the transport of free fatty acid (FFA) into the mitochondria for undergoing mitochondrial fatty acid oxidation (FAO) and finally producing ATP [23]. As shown in Figure 4c,d, the integrated optical intensity corresponding to the level of CPT1A was significantly increased (*p* < 0.05) in the VA-treated group compared to control.

### 3.3. Effect of VA on Embryonic Mitochondrial Function

**Figure 5:**
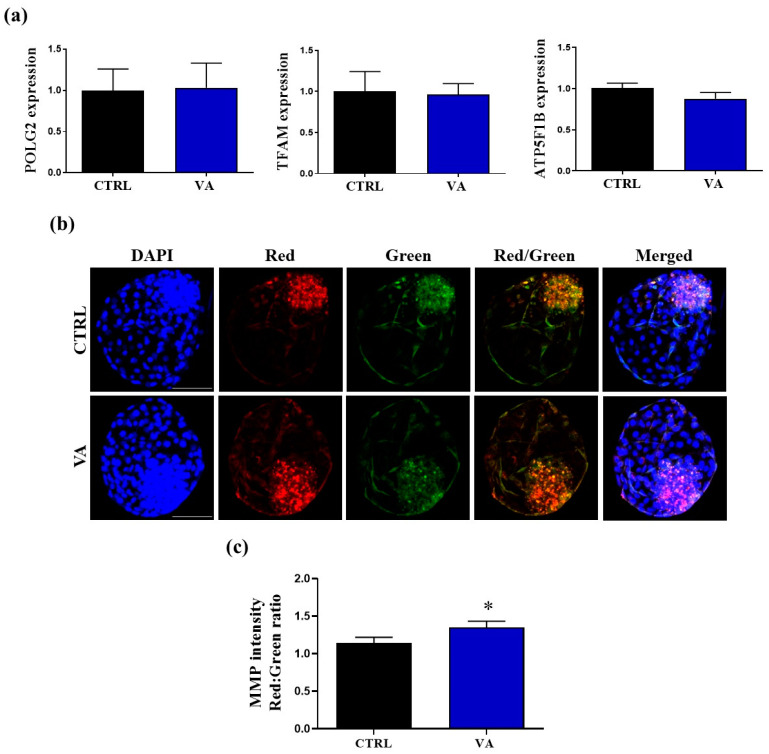
Effect of VA on mitochondrial activity. (**a**) Relative expression of the mRNA of mitochondrial-related genes in day-8 blastocysts. (**b**) Representative images of J-aggregate (red) and J-monomer (green) in day-8 blastocysts. Scale bar, 100 µm. (**c**) Mitochondrial membrane potential (ΔΨ_m_) intensity detected in day-8 blastocysts. *p* value < 0.05 was reported a statistically significance and presented as asterisk (*).

Furthermore, we examined the effect of VA on mitochondrial function through investigating the transcription pattern of certain mitochondrial-function-related genes, as well as analyzing the mitochondrial membrane potential (ΔΨ_m_) in day-8 blastocysts. *POLG2* [24], *TFAM* [25], and *ATP5F1B* [26] have been previously used as indicators for mitochondrial function in bovine oocytes. Although the impact of VA on the mRNA levels of *POLG2*, *TFAM*, and *ATP5F1B* was non-significant, (*p* > 0.05; Figure 5a), the ratio of J-aggregate versus J-monomer was significantly higher (*p* < 0.05) in the VA group compared to control (Figure 5b,c), indicating that VA can improve the mitochondrial membrane potential (ΔΨ_m_).

### 3.4. Regulation of Redox Homeostasis and Apoptosis in VA-Treated Embryos

**Figure 6:**
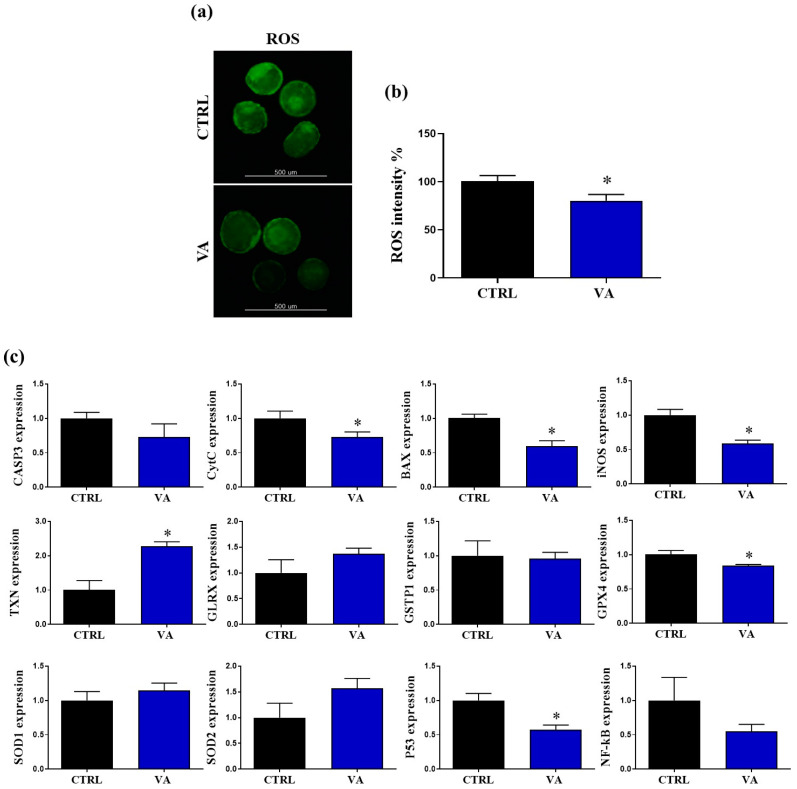
Effect of VA on the oxidative stress and redox homeostasis in blastocysts. (**a**) Microscopic determination of day-8 blastocysts stained with H_2_DCFDA, an indicator for intracellular ROS. Scale bar, 500 µm. (**b**) Fluorescent intensity of ROS signal in VA-treated and control groups. (**c**) Relative expression of the mRNA of oxidative-stress- and homeostasis-related genes in day-8 blastocysts. *p* value < 0.05 was reported a statistically significance and presented as asterisk (*).

Excessive accumulation of ROS impairs mitochondrial function and hence initiates further release of ROS. We continued to elucidate the impact of VA on apoptosis and redox homeostasis in bovine embryos through investigating the ROS levels in day-8 blastocysts. Importantly, lower florescence signals, corresponding to ROS activity, were detected in the VA-treated group compared to the control (*p* < 0.05; Figure 6a,b).

Additionally, we measured the expression levels of the critical markers regulating redox homeostasis and oxidative stress in blastocysts at the mRNA level. As seen in Figure 6c, the RT-qPCR results showed significant down-regulation of the apoptosis-related genes *P53*, *BAX*, *Cytc*, and *iNOS* in VA-treated embryos (*p* < 0.05). On the other hand, the mRNA level of the redox homeostasis-related gene *TXN* showed significant up-regulation in VA-treated embryos compared with the control group (Figure 6c).

### 3.5. Regulation of AKT Signaling in VA-Treated Embryos

**Figure 7:**
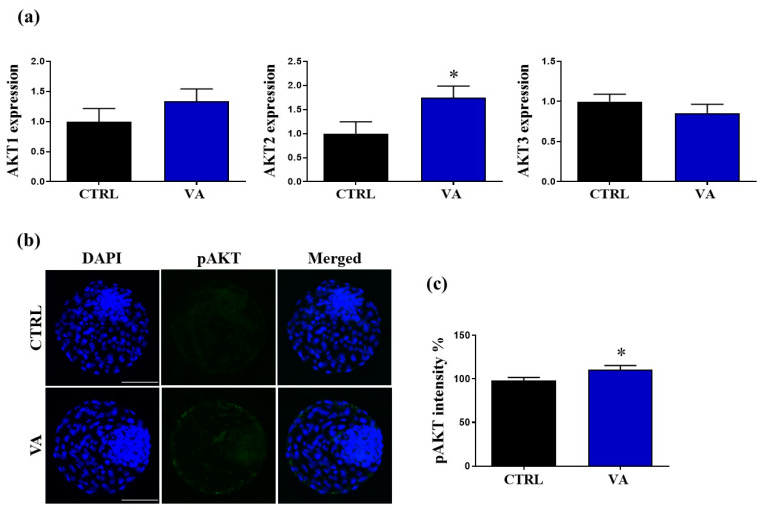
Effect of VA supplementation on the AKT signaling in blastocysts. (**a**) The relative expression of the mRNA of the AKT-signaling-related genes (AKT1, AKT2, and AKT3) in day-8 blastocysts. (**b**) Representative images of day-8 blastocysts stained with phosphorylated AKT-Ser473 (pAKT-Ser473) antibody. Scale bar, 100 µm. (**c**) Mean values of the integrated optimal density of pAKT-Ser473 in VA-treated and control blastocysts. *p* value < 0.05 was reported a statistically significance and presented as asterisk (*).

Finally, to decipher the potential mechanisms behind the beneficial effect of VA treatment on the in vitro developmental competence of bovine embryos, the transcriptional and translational patterns of AKT were investigated. The results of RT-qPCR showed significant up-regulation of *AKT2* in the VA-treated group (*p* < 0.05), whereas *AKT1* and *AKT3* did not show any significant difference compared to the untreated control (Figure 7a). In addition, testing the protein level of phosphorylated AKT (pAKT-Ser473) showed significant overexpression in the VA-treated group compared to the untreated control (Figure 7b,c).

## 4. Discussion

The use of assisted reproductive technologies (ART) in both human and livestock reproduction has undergone considerable changes over the last decade. Excessive exposure of the oocytes/embryos to light and high oxygen tension in vitro usually results in disturbances in redox equilibrium and increases the production of free radicals [27]. Dysregulation of redox status during oxidative stress causes mitochondrial dysfunction, RNA/DNA and protein damage, and induction of blastomere apoptosis [1]. Efforts have revealed the critical need to supplement the propagation media with certain concentrations of antioxidants to effectively control the oxidative-stress-induced embryo loss [28,29,30]. The antioxidant potential of VA has been previously reported in different mammalian cells [13,17,18]. Nevertheless, its effect during embryo development has not been yet elucidated. In the present study, we investigated the antioxidant potential of VA supplementation during in vitro maturation and/or late embryo culture on redox homeostasis, mitochondrial function, AKT signaling, and the developmental competence of oocytes and pre-implantation embryos.

Previously, high VA concentrations (200–1000 μM) induced oxidative stress and apoptosis in a NCI-H460 lung cancer cell line [31], while lower concentrations (0.1–100 μM) inhibited ROS overproduction in human umbilical vein endothelial cells (HUVECs), liver BNLCL2, and murine melanoma B16F0 cells [32,33]. In addition, the protective effect of VA (50–200 μM) against β-amyloid-induced neurotoxicity has been reported in neuronal HT22 cells [17]. Using a wide range of VA concentrations ranging from 2–2000 μM, our initial data showed that the addition of VA during oocyte maturation improved the number of successfully cleaved embryos. Testing the quality of oocytes following VA treatment revealed down-regulation of the apoptosis-related genes *P53*, *BAX*, and *CASP3*. Matching with our finding, the active form of *CASP3* has been recognized in human oocytes with low developmental competence; oocytes that failed to cleave following fertilization [34]. Similarly, the pro-apoptotic gene *BAX* is a positive regulator of apoptosis and its lower expression may contribute to the reduced apoptosis of cells [35], whereas the transcriptional activity of tumor suppressor p53 protein is linked to cell death [36]. The results suggested a relationship between VA-improved oocyte developmental competence and altered transcriptional activities of apoptosis-related genes.

Afterward, dual administration of VA during IVM and late embryo culturing significantly improved the blastocyst development rate as well as the quality of embryos on term of total number of cells and TE per blastocyst. Based on cell number and cohesion, the morphology of the TE and the ICM has been widely used as a scoring system to grade human blastocysts before transfer [37]. Recent investigations emphasized the importance of TE morphology for pregnancy outcomes, since ICM cannot further develop without TE establishing implantation [38]. Additionally, total cell number provides a significant additional information for embryo quality, as embryos with a higher number of cells were reported to be more likely to implant and develop into live offspring [39,40]. Since the distortions of apoptosis can lead to early embryonic death or fetal anomalies [41], we measured the expression levels of apoptosis-specific markers in day-8 blastocyst. The observed down-regulation of the apoptosis-related genes in VA-treated embryos is consistent with the early findings showing altered transcriptional activities of *BAX* and *P53* genes in mouse zygotes following droplet vitrification [36].

Since ROS and free radicals are highly reactive, they can potentially damage DNA and proteins and induce lipid peroxidation in organisms [33]. VA exerted a specific anti-oxidant effect on memory and learning in mice and abolished the detrimental effect of oxidative stress [42]. The VA antioxidant effect was accomplished through the induction of Akt, Nrf2, and GSK-3β signaling in the brains of mice [43]. Additionally, vanillin was reported to inhibit lipid peroxidation and protein oxidation in the mitochondria of rat liver [33]. To demonstrate the antioxidant effects of VA on redox homeostasis in bovine embryos, the ROS level was estimated in day-8 blastocysts. The lower ROS-specific fluorescence signal detected in VA-treated embryos was further confirmed by RT-qPCR, which showed up-regulation of the redox homeostasis-related gene *TXN* in VA-treated embryos, reflecting a role of VA in reducing oxidative stress. Previously, it has been reported that mitochondrial membrane potential could be an obvious indicator for embryo viability [44,45], yet low mitochondrial activity may be linked to feeble elimination of the ROS from the cells [46,47]. Consistent with this, we observed that VA supplementation can improve the mitochondrial membrane potential and reduce ROS generation. These results are supported by the previous study reporting that VA reduced the production of ROS, restored mitochondrial membrane potential, and improved the activities of antioxidant enzymes in human umbilical vein endothelial cells [32].

Fatty acid oxidation is a metabolic process responsible for the production of ATP, which is essential for cell proliferation event in pre-implantation embryos [48]. Surprisingly, the imbalance in the redox state can impair lipid metabolism, leading to accumulation of intracellular lipids [49]. The main consequence of ROS is the accumulation of lipid droplets which are sequentially peroxidated in the presence of ROS [50]. The lipid droplets accumulation occurs in mutant mice with neuronal mitochondrial dysfunction, indicating that lipid droplet formation upon oxidative stress represents an early indicator and promoter of neurodegenerative disease [50]. In the present study, the accumulated lipid content was lower in VA-treated embryos, confirming the effects of VA on redox homeostasis. The ability of VA to inhibit lipid accumulation in mice adipocytes was previously reported in vitro and in vivo [15], which supports our finding. For more confirmation, the expression of mitochondrial enzyme carnitine palmitoyl transferase 1A (CPT1A) was estimated by immunofluorescence. The CPT1A constitutes a rate-limiting step of fatty acid oxidation through controlling the entry of long-chain fatty acids into mitochondria [51]. In our study, VA treatment increased the level of CPT1A in embryos, suggesting that the lower lipid content might be due to high fatty acid oxidation metabolism in response to VA-induced CPT1A activity.

The active role of AKT signaling in the context of embryo development has been previously documented [11,30]. However, no data are available on the possible linkage between VA and AKT signaling in mammalian embryos, albeit reported in other cells such as the prefrontal cortex, neuronal HT22 cells, and dermal papilla cells [17,19,20]. For this reason, we investigated the expression levels of AKT at both mRNA and protein levels in VA-treated embryos. Interestingly, the mRNA level of *AKT2* showed significant up-regulation while the pAKT-Ser473 was more abundant in VA-treated embryos. These results are in line with previous studies reporting that VA significantly increased the levels of pAKT-Ser473 in dermal papilla cells and prefrontal cortex of mice [19,20].

## 5. Conclusions

**Figure 8:**
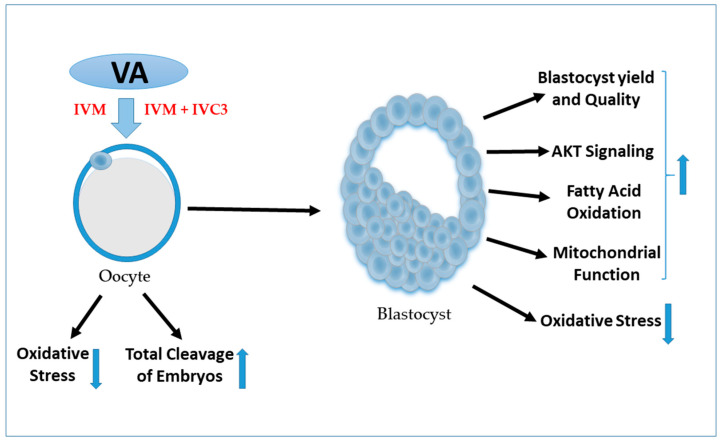
Diagram showing the effect of VA on oocyte and blastocyst development.

To sum up, the dual treatment of VA during bovine oocyte maturation and late embryo culturing stage can improve the developmental competence and the quality of pre-implantation embryos through reducing the oxidative stress, accelerating the fatty acid oxidation, and improving the mitochondrial membrane potential. The systematic diagram for the impact of VA on oocytes and embryos is summarized in Figure 8. This study revealed, as far as we know, for the first time the potential link between VA and the AKT signaling pathway from which affirmative influences on the embryo developmental competence and quality have been recognized. This approach paves the way for the development of more efficacious protocols for use in ART to improve human fertility.

## Figures and Tables

**Table 1:** The names and sequences of the genes/primers used in RT-qPCR analysis.

Names	Primer Sequences	
	Forward	Reverse
P53	CTATGAGATGTTCCGAGAGC	CTCTCTCTTGAGCATTGGTT
BAX	CACCAAGAAGCTGAGCGAGTGT	TCGGAAAAAGACCTCTCGGGGA
iNOS	CGAGCTTCTACCTCAAGCTATC	CTGGCCAGATGTTCCTCTATTT
Caspase-3	CCCAAGTGTGACCACTGAAC	CCATTAGGCCACACTCACTG
Cytc	CCAGGTAGCCAAGGATGTGT	CTTTCGGCTCTTGAGGACTG
BCL2	TGGATGACCGAGTACCTGAA	CAGCCAGGAGAAATCAAACA
GSTP1	CTCACGCTGTACCAGTCCAA	GCAGCGAAGGTCCTCTACAC
GLRX	CAGAACGGTACCTCGGGTCT	TGCCATCCTATCCCCAAGGG
GPX4	GCACGAATTTTCAGCCAAGG	AAACCACACTCGGCGTATCG
NF-kB	TGGCGGAATTACCTTCCATAC	CATCACTCTTGCCACAACTTTC
SOD1	CCATCCACTTCGAGGCAAAG	TCTCCAAACTGATGGACGTGG
SOD2	GGGAGAATGTAACTGCACGA	ACAACAGAGCAGCGTACTGG
TXN	TCGGATCCGTGTCCATCGAT	CACGTGGCTGAGAAGTCGAC
SIRT1	CAACGGTTTCCATTCGTGTG	GTTCGAGGATCTGTGCCAAT
POLG2	CTTCTGGGAAACTACGGGAGAAC	GTAGCCTCTTGTTTACCAGATCCA
TFAM	CTGGTCAGTGCTTTGTCTGC	CTAAAGGGATAGCGCAGTCG
ATP5F1B	TGCTTTATTGGGCAGAATCC	GATCCGTCAAGTCATCAGCA
AKT1	AAAAGGAAGTGGTGTACAGG	GAAGTCGGTGATCTTGATGT
AKT2	CGACTATCTCAAACTCCTGG	ATCTTCATGGCATAGTAGCG
AKT3	AGCTGTTTTTCCATTTGTCG	TGTAGATAGTCCAAGGCAGA
GADPH	CCCAGAATATCATCCCTGCT	CTGCTTCACCACCTTCTTGA

## Data Availability

All the data used to support the findings of the current study are included within the article.
